# Snoring and apnoea linked to higher stroke risk: US study

**Published:** 2023

Experts have found that people with the common sleep disorder apnoea, which has hundreds of millions of sufferers worldwide, are five times likelier to develop atrial fibrillation. They said that a number of people with apnoea, symptoms of which include stopping and starting breathing, making snorting noises, waking up a lot and loud snoring, can go undiagnosed, reports The Guardian.

The condition is already known to heighten the risk of serious health problems such as high blood pressure, type 2 diabetes and depression, but now the US researchers have uncovered even more evidence about its impact on the heart, saying it significantly raises the risk of atrial fibrillation and stroke. The findings were discussed by doctors at the annual meeting of the European Society of Cardiology, the world’s largest heart conference, in Amsterdam, reports The Guardian.

Studies involving experts from Stanford University looked at about 1.7 million people aged 20 to 50 years over a decade. Those with sleep apnoea were five times more likely to develop atrial fibrillation and 60% more likely to experience a stroke later in life, they found.

Sanjiv Narayan, a professor of cardiovascular medicine at Stanford and the study’s author, said: ‘We found a 60% increased risk of having a stroke if you have sleep apnoea. The condition is really common but we tend to ignore it because we think it’s trivial or just a nuisance. Until now, no one’s really shown the magnitude of the size of the risk. That’s what really surprised us – and also this is in the relatively young.’

Sleep apnoea happens if your airways become too narrow while you sleep. This stops you breathing properly. Its causes are not always clear, but it has been linked to factors such as obesity, having a large neck, smoking and drinking alcohol, and sleeping on your back.

Atrial fibrillation causes an irregular and often abnormally fast heart rate.

Narayan said: ‘When you are unable to breathe, it raises the pressure in the lungs until you ultimately wake up gasping for breath. That puts a pressure load on the heart, which causes stretch in the heart chambers, and could cause the atrial fibrillation. Another theory could be that the oxygen levels in the blood fall for tens of seconds, which could put stress on the heart.’
